# A Master Curve for Fatigue Design of Notched Nodular Cast Iron Components Based on the Local Averaged Strain Energy Density

**DOI:** 10.3390/ma17194807

**Published:** 2024-09-29

**Authors:** Jacopo Pelizzari, Alberto Campagnolo, Carlo Dengo, Giovanni Meneghetti

**Affiliations:** 1 Department of Industrial Engineering, University of Padova, Via Venezia 1, 35131 Padova, Italy; jacopo_pelizzari@carraro.com (J.P.); alberto.campagnolo@unipd.it (A.C.); 2 Virtual Validation Department, Carraro S.p.A., Via Olmo 37, 35011 Campodarsego, Italy; carlo_dengo@carraro.com

**Keywords:** nodular cast iron, off-highway axles, fatigue, V-notch, strain energy density

## Abstract

The industry of off-highway vehicles is one of the fields of major application of nodular cast irons, which guarantee the manufacture of complex geometries and ensure good mechanical properties. The present investigation deals with the fatigue design of off-highway axles made of EN-GJS-500-7. Typically, off-highway axles are weakened by stress risers which must be assessed against fatigue. In this investigation, laboratory specimens have been extracted from an off-highway axle to take into account the manufacturing process effects. Different specimens’ geometries have been prepared, including plain, bluntly notched and sharply V-notched specimens, and constant amplitude, load-controlled axial fatigue tests were conducted using two nominal load ratios, namely push–pull and pulsating tension loading. As a result, both the notch and the mean stress effects on the fatigue behaviour of EN-GJS-500-7 have been experimentally investigated for the first time. A well-known local approach, which takes the strain energy density (SED) averaged over a properly defined structural volume as a fatigue damage parameter, has been applied both in the linear elastic and elastic plastic formulations. Since the SED correlated the geometrical notch effects of the specimens as well as the mean stress effects, a master curve based on the averaged SED has been defined for the first time, to the best of the authors’ knowledge, for the fatigue design of off-highway axles made of EN-GJS-500-7.

## 1. Introduction

Off-highway axles are typically manufactured by using nodular cast irons due to their good mechanical properties coupled with net shape casting capability at reduced costs. The fatigue strength assessment of such components is a tricky task due to both the complex geometry, with the presence of several stress concentration regions, and the severe loading conditions, which include variable amplitude, mean stress, overload effects, etc. As a result, structural engineers involved in designing off-highway axles require accurate material strength data and effective fatigue analysis methods.

In this context, the notch effect on the fatigue behaviour of nodular cast irons has been experimentally investigated in several papers [1,2,3,4,5,6] by considering specimens weakened by notches having different severities and made by EN-GJS-400 [3,6], EN-GJS-450 [6], EN-GJS-600 [1,2], EN-GJS-700 [6] and EN-GJS-900 [5] nodular cast irons. The mean stress effect has also been widely investigated in the technical literature by performing experimental fatigue tests under constant amplitude axial loading with different load ratios/mean stresses on specimens made by EN-GJS-400 [3,6,7,8,9,10], EN-GJS-450 [6,10], EN-GJS-700 [6,10] and EN-GJS-900 [5] nodular cast irons. Moreover, the effect of different strain ratios has been experimentally investigated by performing strain-controlled fatigue tests on specimens made by EN-GJS-450 nodular cast iron [11] and austempered ductile irons [12].

Concerning the available criteria for the fatigue assessment of nodular cast iron components, many papers in the technical literature have investigated the effectiveness of stress-life approaches [6,13,14,15,16,17,18] and strain-life approaches [6,19,20,21,22]. These criteria typically include corrections for different load/strain ratios, but they are not capable of effectively taking into account the detrimental effect of sharp V-notches, i.e., with reduced tip radius. To deal with such cases, several local approaches have been proposed in the literature based on the Theory of Critical Distances (TCD) [1,23], the averaged strain energy density (SED) [2,3] or other fracture mechanics-based approaches [24,25]. Among the others, one of the most widely employed is that based on the strain energy density (SED) [26] averaged over a properly defined structural volume. The fatigue approach based on the averaged SED has been validated against experimental results generated by testing notched specimens made of structural steels [27,28,29], aluminium alloys [29], titanium alloys [30,31] and nodular cast irons, namely EN-GJS-400 [3], EN-GJS-450 [32] and EN-GJS-600 [32]. In all previous works, the averaged SED has been shown to correlate the geometrical notch effects as well as the mean stress effects in a material-dependent, master fatigue design curve.

In this work, constant amplitude, load-controlled axial fatigue tests have been performed on specimens extracted from an off-highway axle made of EN-GJS-500-7 nodular cast iron. Different specimens’ geometries, namely plain, sharply V-notched and bluntly notched specimens, have been tested by adopting two stress ratios, i.e., R = −1 and R = 0.05, with the aim of investigating for the first time, to the best of the authors’ knowledge, both the notch and mean stress effects on the fatigue behaviour of EN-GJS-500-7. Finally, the averaged SED approach has been applied, both in the linear elastic and elastic plastic formulations, to the new experimental fatigue results with the aim of defining a new master curve for the design of off-highway axles made of EN-GJS-500-7. To the best of the authors’ knowledge, no papers in the literature have been devoted to investigating the notch and mean stress effects on the fatigue strength of EN-GJS-500-7 nodular cast iron; moreover, a fatigue design master curve based on the averaged SED has never been calibrated for such material. The shortcomings of the existing research, which the present work aims to reduce, can be summarised as follows:

- The lack of experimental investigations on the notch and mean stress effects on the fatigue behaviour of EN-GJS-500-7 nodular cast iron.
- The lack of a master curve for fatigue design of structural components made of EN-GJS-500-7 nodular cast iron, regardless of the geometry and the loading condition, based on a damage parameter that is relatively easy to calculate.

## 2. Theoretical Background: The Averaged Strain Energy Density (SED) Approach

**Figure 1:**
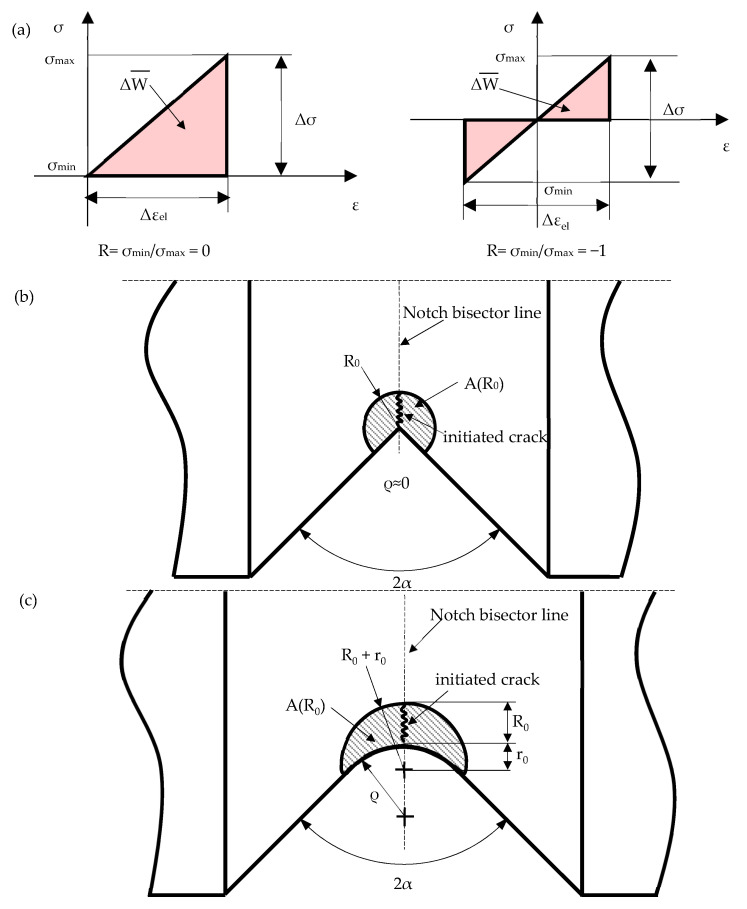
(**a**) Strain energy density with nominal load ratios R = 0 and R = −1. Control volume to calculate the averaged SED for specimens with (**b**) a sharp or (**c**) a blunt notch.

Lazzarin and Zambardi [26] originally proposed the linear elastic strain energy density (SED), $\Delta \bar{W}$ (see Figure 1a), averaged over a structural volume that surrounds the crack initiation location (see Figure 1b,c) as a local parameter to correlate the fatigue strength of notched components. Lazzarin and Zambardi took inspiration from the fundamental works by Neuber [33,34,35], Peterson [36], Tanaka [37], Sheppard [38] and Taylor [39], who first proposed the calculation of average stress over a material-dependent microstructural length or volume to deal with the fatigue behaviour of notched components.

When dealing with plain specimens under pure axial loading, Equation (1) allows the calculation of $\Delta \bar{W}$ as a function of the nominal stress range Δσ (see Figure 1a):

(1) $$\Delta \bar{W}=c_{W}\frac{\Delta \sigma \cdot \Delta \varepsilon_{el}}{2}=c_{W}\frac{\Delta \sigma^{2}}{2E}$$

In the previous expression, Δε_el_ is the elastic strain range, E is the Young’s modulus of the material, and the coefficient c_w_ takes into account the nominal load ratio R (see examples in Figure 1a), which is defined by Equation (2) [40].

(2) $$c_{w}=\left\{\begin{array}{c} \frac{1+R^{2}}{{\left(1-R\right)}^{2}}\;\mathrm{if}\;-1\leq R\leq 0\\ \frac{1-R^{2}}{{\left(1-R\right)}^{2}}\;\mathrm{if}\;0\leq R<1 \end{array}\right.$$

Concerning notched components under pure axial loading, the calculation of $\Delta \bar{W}$ for the case of notches having null tip radius (ρ ≈ 0) has been theoretically formalised by Lazzarin and Zambardi [26], while the case of notches with a tip radius greater than zero has been treated later on by Lazzarin and Berto [41].

Based on such works [26,41], the structural volume inside which the SED is averaged needs to be geometrically defined as follows:

- For notches with tip radius ρ ≈ 0, the structural volume is centred at the notch tip and has a circular shape with radius R_0_ [26] (see Figure 1b, A(R_0_) being the area of the structural volume);
- For notches with tip radius ρ > 0, Lazzarin and Berto [41] suggested a structural volume having a crescent shape (see Figure 1c, A(R_0_) being the area of the structural volume). Here, R_0_ measures the depth of the crescent shape along the notch bisector line. Such a structural volume can be obtained by modelling a circular shape having a radius equal to (R_0_ + r_0_) and centre at a distance r_0_ behind the notch tip. The parameter r_0_ is defined by Equation (3):
  (3) $$r_{0}\;=\;\frac{q-1}{q}\rho \;\mathrm{where}\;q\;=\;\frac{2\pi -2\alpha }{\pi }$$
  where 2α is the notch opening angle and ρ the notch tip radius.

According to Lazzarin and co-workers [26,28,42], the structural volume size, R_0_, is thought to be a property of the material and loading type; moreover, it can be calibrated by equating the averaged SED at the fatigue limit condition or the high-cycle fatigue strength of two opposite geometrical configurations, namely the plain and the sharp notch (ρ ≈ 0) geometries.

Once the structural volume shape and size are properly defined, the averaged SED for notched specimens, $\Delta \bar{W}$, can be calculated by directly post-processing the results of linear elastic FE analyses, according to the so-called “direct approach” expressed by Equation (4).

(4) $$\Delta \bar{W}=c_{W}\frac{\sum_{i\in A(R_{0})}\left[{\left.\left(\frac{\Delta \sigma_{jk,i}\cdot \Delta \varepsilon_{jk,el,i}}{2}\right)\right|}_{j,k=x,y,z}\cdot A_{i}\right]}{\sum_{i\in A(R_{0})}A_{i}}$$

In the previous expression, Δσ_jk,i_ and Δε_jk,el,i_ are the ranges of the *jk*-th components (j, k = x, y, z in a Cartesian reference system) of the stress and elastic strain, respectively, referring to the *i*-th finite element having area A_i_ and belonging to the structural volume of radius R_0_. The strain energy density contribution of all stress–strain components is included in Equation (4) through the indexes *j* and *k*, since the application of a pure axial loading to a notched cylindrical specimen generates not only axial but also hoop stress–strain components close to the notch tip. Finally, c_W_ properly accounts for the nominal load ratio R according to Equation (2), provided that the range value of the load (ΔF, or equivalently Δσ) has been applied to the FE model. It is worth noting that the term ${\left.\left(\frac{\Delta \sigma_{jk,i}\cdot \Delta \varepsilon_{jk,el,i}}{2}\right)\right|}_{j,k=x,y,z}\cdot A_{i}$ in Equation (4) represents the elastic strain energy of the *i*-th finite element, and it is typically available in the post-processing environment of commercial FE codes together with the element area A_i_; e.g., in Ansys^®^ FE code, they are “*element tables*” named “*SENE*” and “*VOLU*”, respectively. This is the reason why Equation (4) is called “direct approach”. Lazzarin et al. [43] demonstrated that coarse mesh patterns can be employed within the structural volume when applying such an approach to calculate the averaged SED.

**Figure 2:**
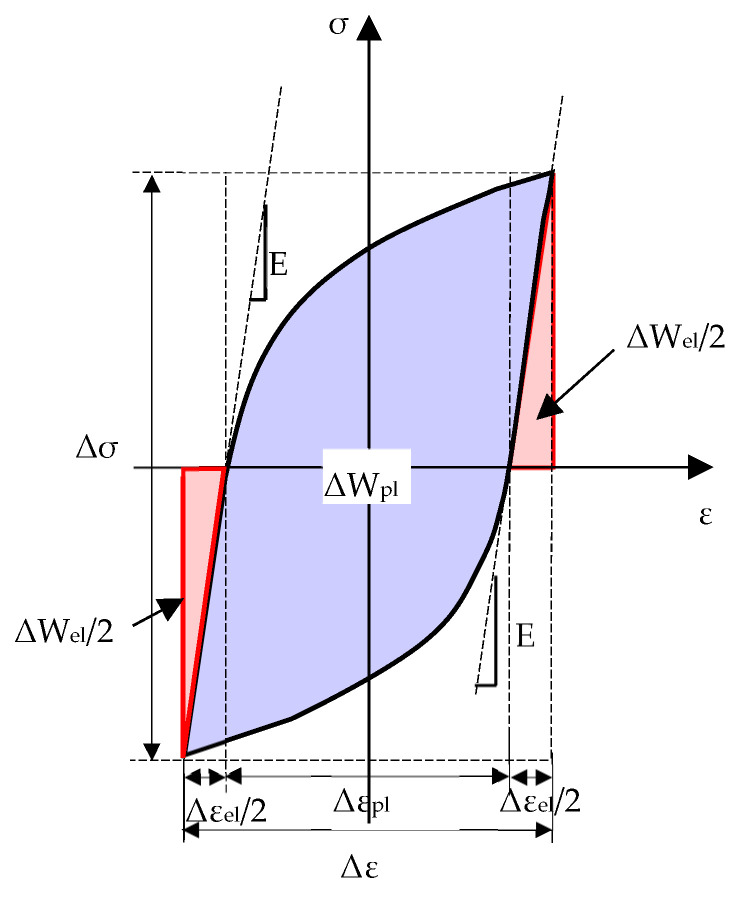
Elastic and plastic strain energy densities for a plain specimen under a nominal load ratio R = −1.

The assumption of a linear elastic material behaviour (see Figure 1a) is typically valid only in the high-cycle fatigue regime of ductile metals; plastic deformations are not negligible when dealing with the low–medium cycle fatigue regime or in the presence of a sharp notch, which introduces severe, local stress and strain concentrations. In latter cases, the cyclic loading generates an accumulation and release of the elastic strain energy as well as dissipation of the plastic strain energy, as defined in Figure 2. Several researchers have proposed fatigue approaches based on plastic strain energy or a combination of elastic and plastic strain energy contributions; among others, the works by Morrow [44] and Ellyin [45,46] deserve to be mentioned.

In this context, the averaged SED approach as proposed by Lazzarin and Zambardi has already been extended to account for an elastic plastic material behaviour in the recent works by Benedetti et al. [29], Schuscha et al. [47], Zhao et al. [48] and Horvath et al. [49]. In more detail, when dealing with plain specimens, the elastic plastic averaged SED can be calculated as follows:

(5) $$\Delta \bar{W}=\Delta W_{el}+\Delta W_{pl}=c_{W}\frac{\Delta \sigma^{2}}{2E}+\oint \sigma d\varepsilon$$

where the elastic contribution (ΔW_el_) keeps the same as in Equation (1), while the plastic strain energy density (ΔW_pl_) is defined as the area comprised within the stabilised hysteresis loop (see Figure 2).

**Figure 3:**
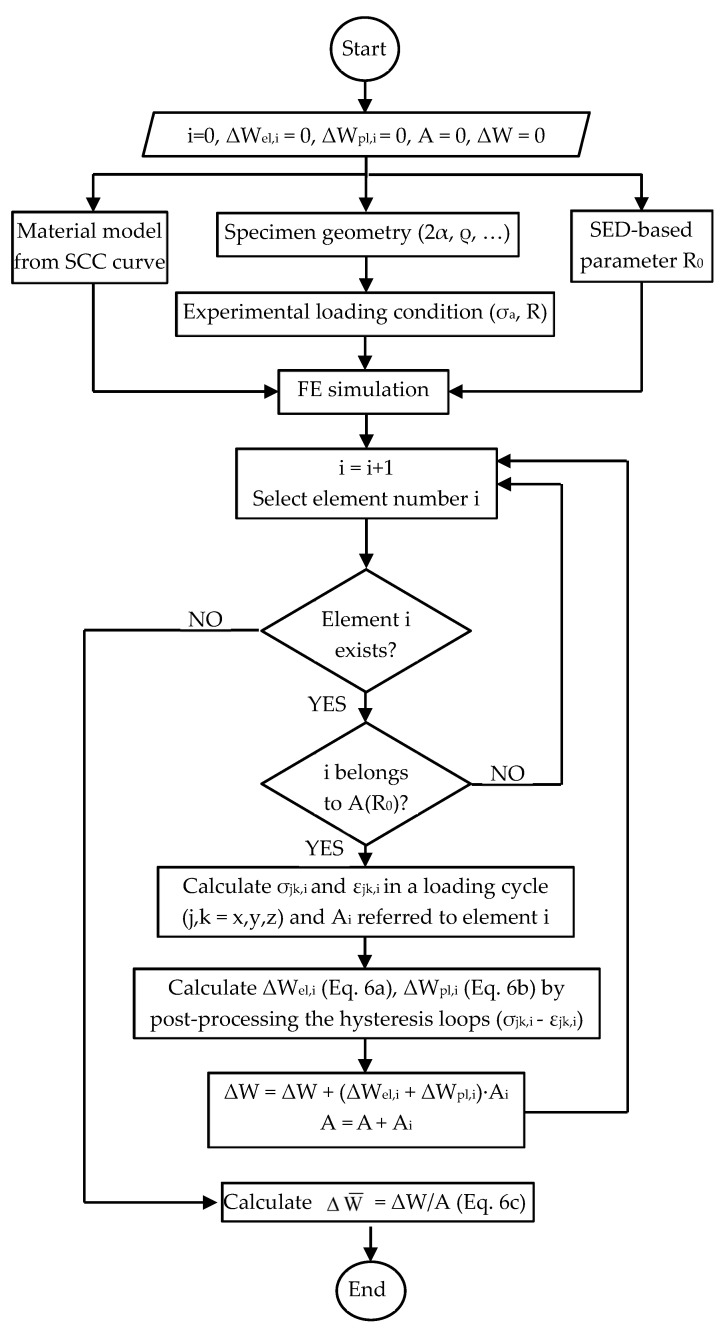
Flowchart of the procedure to calculate the elastic plastic averaged strain energy density.

In the case of notched components, the shape of the structural volume as well as the calibration procedure of the size R_0_ are assumed to be the same presented above for the linear elastic formulation of the averaged SED approach. On the other hand, Equation (4) must be updated as follows to allow the calculation of the averaged SED under elastic plastic conditions (see also the flowchart reported in Figure 3):

(6a) $$\Delta W_{el,i}=c_{W,jk,i}\cdot \frac{\Delta \sigma_{jk,i}\cdot \Delta \varepsilon_{jk,el,i}}{2}\begin{array}{c} \;\mathrm{where}\; \end{array}c_{W,jk,i}=f\left(R_{jk,i}=\frac{\sigma_{jk,i,\min}}{\sigma_{jk,i,\max}}\right)$$

(6b) $$\Delta W_{pl,i}=\oint \sigma_{jk,i}d\varepsilon_{jk,i}$$

(6c) $$\Delta \bar{W}=\frac{\sum_{i\in A(R_{0})}\left[\left(\Delta W_{el,i}+\Delta W_{pl,i}\right)\cdot A_{i}\right]}{\sum_{i\in A(R_{0})}A_{i}}$$

Equation (6) shows that the elastic plastic averaged SED for notched components includes the coefficient $c_{W,jk,i}$, which is still defined by Equation (2) but it accounts for the local stress ratio R_jk,i_ for each *jk*-th stress component (j, k = x, y, z in a Cartesian reference system) and referred to the *i*-th finite element. This allows the case of notched components to be subjected to axial loading with nominal load ratio R ≠ −1 under small scale yielding. For example, in the case R = 0, plastic deformations localised at the notch tip shift the local stress ratio R_jk,i_ to negative values, while the material region far from the notch tip, where plastic deformations are negligible, keeps a local stress ratio equal to the nominal load ratio.

It is worth noting that Equation (6) requires that the experimental load or stress cycle is properly simulated in the FE analysis aimed at calculating the averaged SED, the material behaviour being elastic plastic and, therefore, sensitive to the load path. Moreover, Equation (6) cannot be considered as a “direct approach”, as is Equation (4), since to be applied, it requires for each *i*-th finite element belonging to the structural volume:

- Calculation of the hysteresis loops σ_jk_-ε_jk_ in the post-processing environment of the FE code with reference to one stress cycle.
- Calculation of the elastic and plastic strain energy density contributions, i.e., $\Delta W_{el,i}$ and $\Delta W_{pl,i}$, by post-processing the hysteresis loops, e.g., by taking advantage of a dedicated numerical code.

## 3. Materials and Methods

### 3.1. Specimens’ Position

The fatigue behaviour of a pearlitic nodular cast iron, EN-GJS-500-7 [50], has been investigated in the present work. Specimens have been extracted from an axle’s trumpet with the aim of taking into account the effects of the manufacturing processes on the material fatigue strength. The geometry of the axle’s trumpet along with the detailed specimens’ locations have already been presented in a previous work [51], to which the reader is referred. More precisely, the specimens tested here have been extracted from the locations highlighted in Figure 1 of Ref. [51] and referred to ‘*specimens for strain-controlled fatigue tests*’. The geometries of the tested specimens will be presented in Section 3.3.

### 3.2. Material Microstructure, Hardness, Static and Strain-Controlled Fatigue Properties

The same axle’s trumpet considered here has been investigated in a previous work [51], which focused on the static and strain-controlled fatigue characterisation of the EN-GJS-500-7 nodular cast iron. The main outcomes are summarised here.

**Figure 4:**
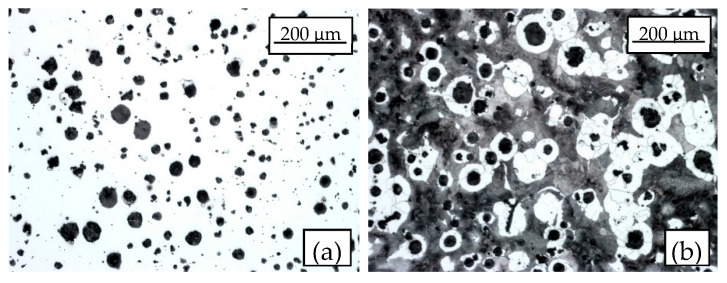
Microstructures of the EN-GJS-500-7 pearlitic cast iron (**a**) before and (**b**) after Nital 2% etching. The microstructure is shown at 100× magnification according to ISO 945:1 2019. Reprinted with permission from Ref. [51]. 2024, Elsevier.

According to the metallographic analyses reported in Figure 4, the graphite nodules have been classified as VI—6/7 [52], and the matrix has been shown to be pearlitic for the 60% and ferritic for the 40%. The Brinell hardness [53] has resulted in a range of 220–230 HBW. A link between the matrix microstructure along with the graphite nodule characteristics and the fatigue strength of a nodular cast iron can be established thanks to the Murakami approach [54]. Accordingly, the fatigue limit can be estimated on the basis of the Vickers hardness of the matrix (HV_matrix_) and the $\sqrt{area}$ parameter, defined as the maximum size of the graphite nodules evaluated with the extreme value statistics.

The main results of the static tensile tests [55] and strain-controlled fatigue tests [56] conducted at room temperature in Ref. [51] have been summarised in Table 1. Dealing with the static tensile curve, Table 1 reports: the modulus of elasticity (E), the proof stress (σ_p0.2_), the tensile strength (σ_R_), the elongation after fracture (A%), and the reduction of area (Z%) as well as the Ramberg-Osgood parameters, i.e., the strength coefficient (K) and the strain hardening exponent (n). Table 1 also includes the parameters of the stabilised cyclic curve (SCC), as derived from strain-controlled fatigue tests: the cyclic strength coefficient (K′) and the cyclic strain hardening exponent (n′).

### 3.3. Constant Amplitude, Load-Controlled Fatigue Testing: Parameters and Specimens’ Geometry

**Figure 5:**
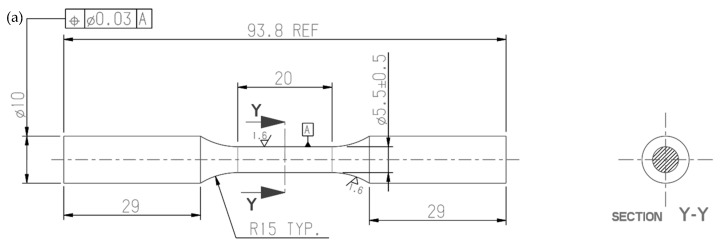

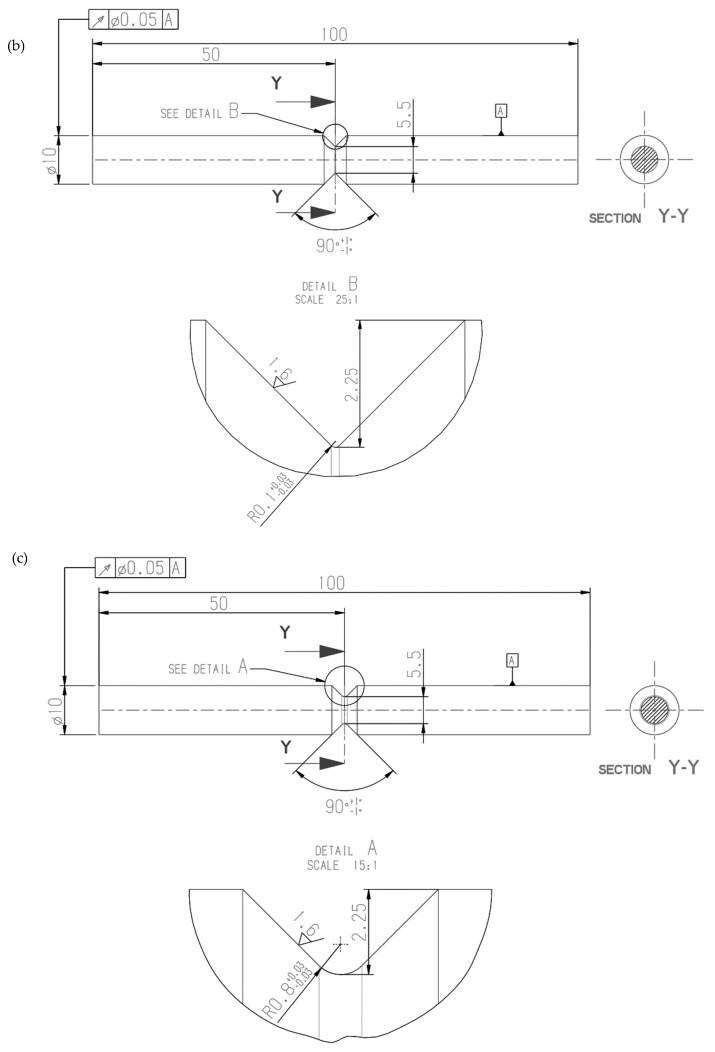
Geometry of (**a**) plain specimen, (**b**) sharply V-notched specimen and (**c**) bluntly notched specimen.

Constant amplitude, load-controlled axial fatigue tests have been conducted on cylindrical specimens extracted from the axle’s trumpet shown in [51] and made of EN-GJS-500-7 nodular cast iron. Both plain (Figure 5a) and notched specimens (Figure 5b,c) have been tested in order to investigate the fatigue notch effect. In more detail, a notch having an opening angle 2α = 90° and a notch tip radius ρ = 0.1 mm (sharp V-notch, Figure 5b) or ρ = 0.8 mm (blunt notch, Figure 5c) has been introduced in the cylindrical specimens to reproduce the geometrical features typical of the axle’s trumpet.

The axial fatigue tests have been conducted using an MTS 858 MiniBionix II servo-hydraulic testing machine (MTS, Eden Prairie, MN, USA), with a load capacity of 15 kN and equipped by an MTS TestStar IIm controller. A constant amplitude load cycle under closed-loop load control has been applied with sinusoidal shape and frequency between 15 and 32 Hz depending on the specimen geometry and the load level. The nominal load ratio R, i.e., $\sigma_{\min}/\sigma_{\max}$, has been imposed equal to −1 for plain and sharply V-notched specimens, while a ratio equal to 0.05 has been adopted for all specimens’ geometries. The specimen complete separation has been assumed as failure criterion to stop the test and define the number of cycles to failure N_f_, while run out condition was set at 2 million cycles if failure did not occur. The net-section nominal stress amplitude σ_a_ has been calculated from Equation (7), as a function of the load amplitude *F_a_* and the diameter of the net-section d_net_, being equal to 5.5 mm for all specimen geometries (see Figure 5).

(7) $$\sigma_{a}=\frac{F_{a}}{A_{net}}=\frac{F_{a}}{\frac{\pi }{4}\cdot d_{net}^{2}}$$

After the fatigue tests, the fracture surfaces have been analysed by using a Leica Cambridge 440 Scanning Electron Microscope (SEM) (Leica, Wetzlar, Germany).

### 3.4. Finite Element Analyses to Calculate the Averaged SED

Two-dimensional structural finite element analyses have been performed for each specimen geometry by means of Ansys^®^ 2024 R1 software in order to apply the averaged SED approach recalled in Section 2.

**Figure 6:**
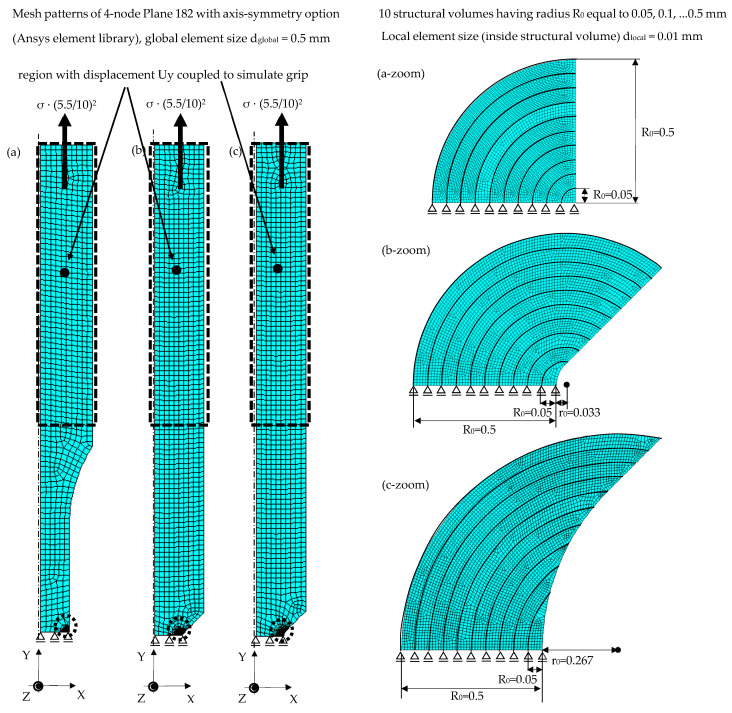
Structural FE analyses to evaluate the averaged SED. FE meshes adopted in the 2D FE models of (**a**) plain, (**b**) sharp V-notched, (**c**) bluntly notched specimens under axial loadings. Dimensions are in millimetres.

Only a quarter of each specimen longitudinal section geometry has been modelled as shown in Figure 6 by taking advantage of the axis symmetry with respect to the Y-axis and the symmetry on the XZ plane. With the size of the structural volume R_0_ not being known a priori, 10 structural volumes having size R_0_ uniformly spaced between 0.05 mm and 0.5 mm have been modelled in each specimen geometry by assuming a circular shape for the plain specimen (Figure 6a) and a crescent shape, as defined in Figure 1c, for the sharply V-notched (Figure 6b) and bluntly notched (Figure 6c) specimens.

A 2D, axis-symmetric, 4-node quadrilateral element (PLANE 182 of Ansys^®^ element library) has been adopted to generate the free mesh pattern reported in Figure 6. A local element size d_local_ = 0.01 mm has been adopted inside the structural volumes, which translates in 20–30 finite elements inside the smallest structural volume (see the zooms in Figure 6), while a global finite element size d_global_ = 0.5 mm has been adopted outside.

Figure 6 highlights also the FE model region where the U_y_ displacement has been coupled to simulate the grip of the testing machine.

The FE models reported in Figure 6 have been solved by assuming two different material behaviours for comparison purposes:

- A *linear elastic material behaviour*: only the modulus of elasticity E (see Table 1) and the Poisson’s ratio (ν = 0.28) have been given as input to the Ansys^®^ 2024 R1 FE software. In such cases, only a single FE model has been solved for each specimen geometry by taking advantage of the linear elasticity and applying an axial stress at the gross section, resulting in a nominal net-section stress σ = 1 MPa.
- An *elastic plastic material behaviour*: the modulus of elasticity E, the Poisson’s ratio (ν = 0.28) and the stabilised cyclic curve (see Table 1) have been given as input to the FE software. A multi-linear kinematic hardening behaviour has been adopted assuming von Mises as the yield criterion. In such cases, an FE model has been solved for each specimen geometry and each load level, with a quadratic proportionality between the averaged SED and the stress level not being applicable. The load has been applied in 100 substeps by simulating two stress cycles with a given nominal stress amplitude σ_a_ and a nominal load ratio R, corresponding to the values adopted in each experimental test.

It is worth noting that FE analyses are not mandatory to calculate the averaged SED for plain specimens since an analytical calculation was possible both for linear elastic (Equation (1)) and elastic plastic (Equation (5)) material behaviour; however, they have been performed for uniformity of the calculation procedure.

## 4. Results

### 4.1. Load-Controlled Fatigue Test: Fracture Surface Analyses

**Figure 7:**
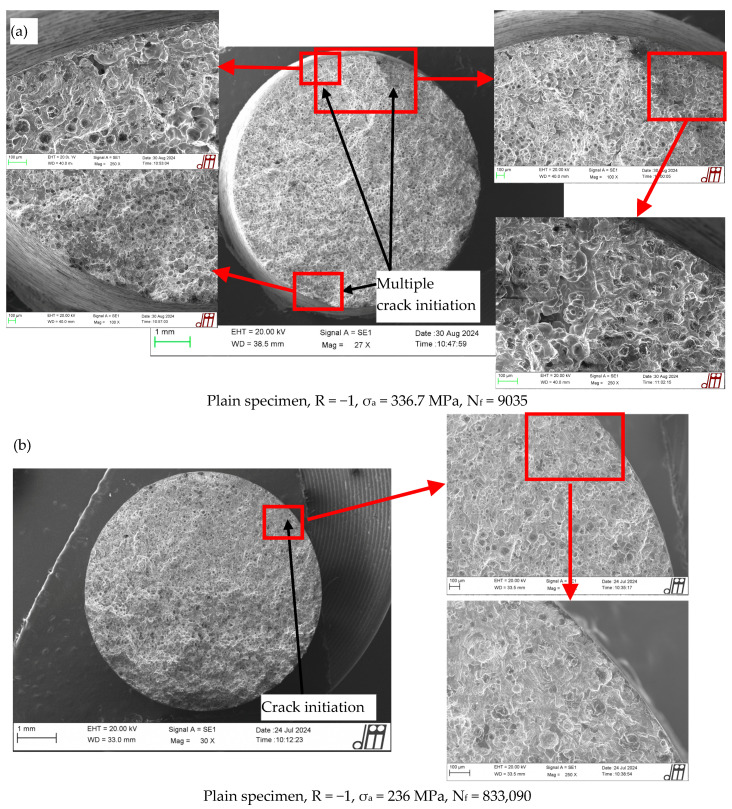

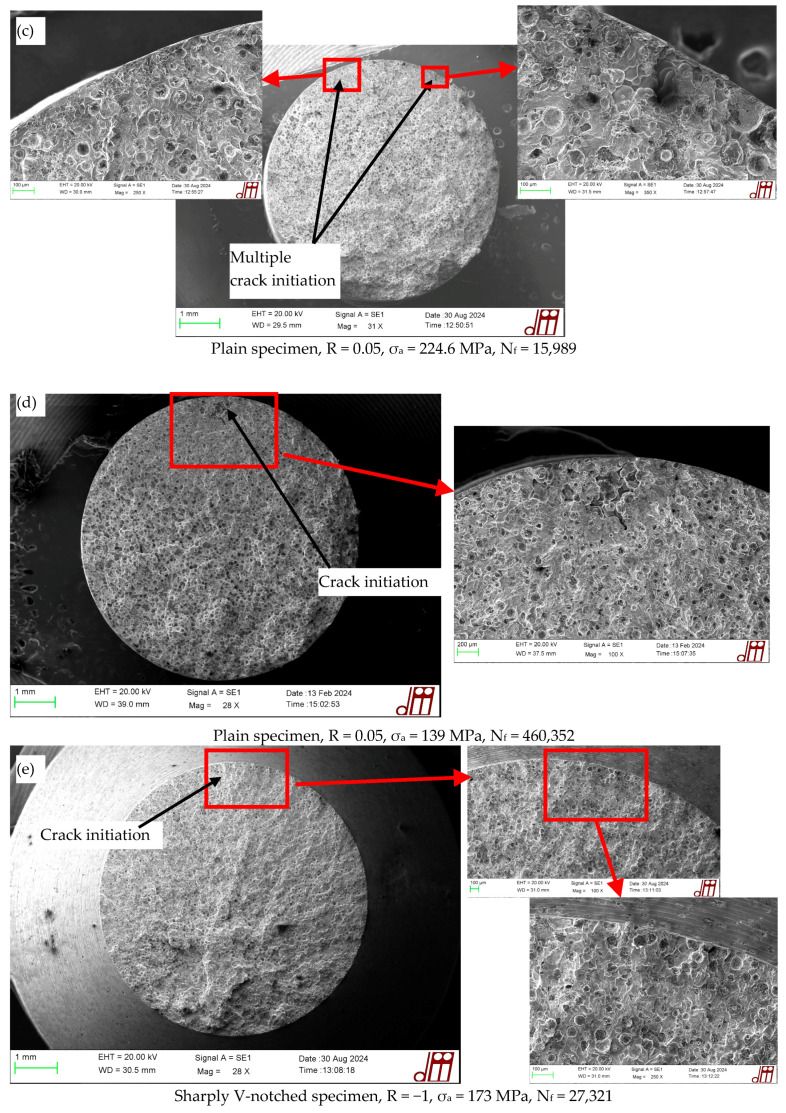

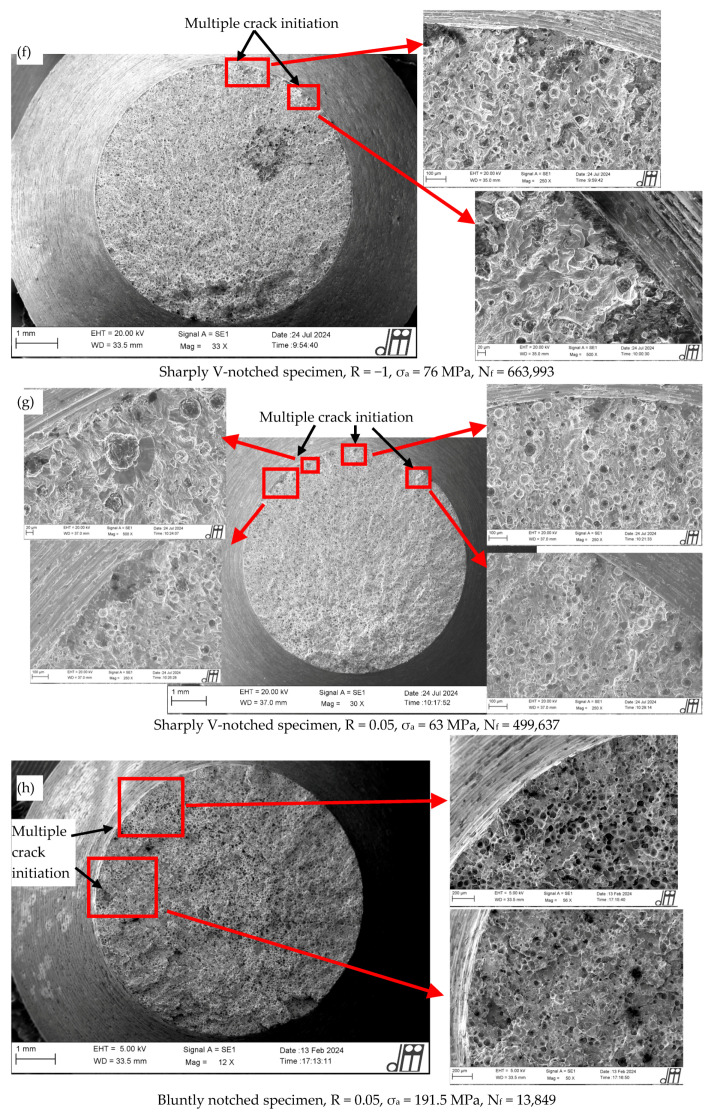

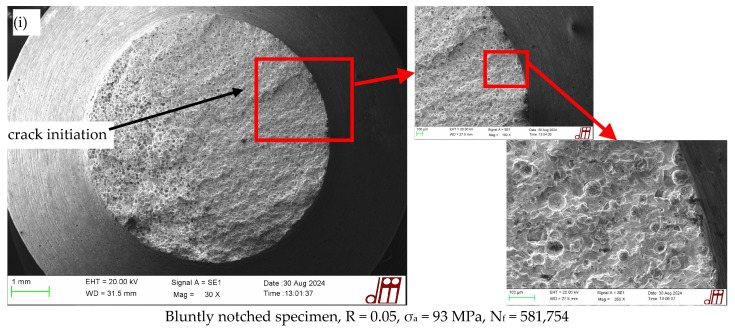
Fracture surfaces after load-controlled axial fatigue tests of plain specimens under (**a**,**b**) R = −1 and (**c**,**d**) R = 0.05; sharply V-notched specimens under (**e**,**f**) R = −1 and (**g**) R = 0.05; bluntly notched specimens under (**h**,**i**) R = 0.05 made of EN-GJS-500-7 cast iron.

The typical fracture surfaces observed after axial fatigue failure of plain, sharply V-notched and bluntly notched specimens have been reported in Figure 7. For each test series, two examples of fracture surfaces have been provided, namely one corresponding to a medium–low number of cycles to failure, and the other one to a high number of cycles to failure. The only exception being the sharply V-notched specimens for which only one fracture surface has been analysed referred to the high-cycle fatigue regime.

When dealing with plain specimens, it can be observed that the EN-GJS-500-7 cast iron exhibited crack initiation either from sub-surface defects (see Figure 7a and Figure 7c,d for R = −1 and 0.05, respectively) or the surface of the specimens (see Figure 7a,b and Figure 7c for R = −1 and 0.05, respectively), with multiple crack initiation locations being visible for the higher load level.

Concerning sharply V-notched specimens, Figure 7e,f for R = −1 and Figure 7g for R = 0.05 show that the fatigue crack initiated from the notch tip both in the medium–low-cycle and high-cycle fatigue regimes, being driven by the dominant notch stress concentration. However, additional crack initiations from sub-surface defects (Figure 7f) and from graphite nodules (Figure 7g) have been observed in the high-cycle fatigue regime.

Dealing with bluntly notched specimens, the crack has initiated from the notch tip both in the medium–low-cycle and high-cycle fatigue regimes as shown in Figure 7h,i, multiple crack initiation locations being visible in both cases, while no defect is evident.

### 4.2. Load-Controlled Fatigue Test: Results

**Figure 8:**
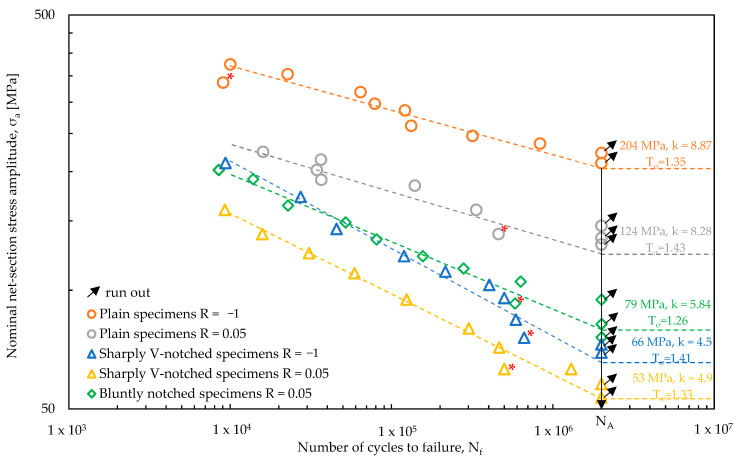
Fatigue test results obtained from plain (Figure 5a), sharply V-notched (Figure 5b) and bluntly notched (Figure 5c) specimens subjected to axial loading are presented in terms of the number of cycles to failure, plotted against the applied nominal net-section stress amplitude.

Figure 8 presents a summary of the experimental data, showing the applied nominal net-section stress amplitude σ_a_ (Equation (7)) plotted against the number of cycles to failure N_f_ and relevant to each test series, namely plain specimens under R = −1 and R = 0.05, sharply V-notched specimens under R = −1 and R = 0.05 and bluntly notched specimens under R = 0.05. A statistical analysis has been performed for each test series according to ISO 12107 [57] to derive the high-cycle fatigue strength σ_A,50%_ with reference to a survival probability of 50% and N_A_ = 2 × 10^6^ cycles, the inverse slope *k* and the scatter index T_σ_, referred to survival probabilities of 10% and 90%, and to a 95% confidence level. For the sake of clarity, Figure 8 reports only the fatigue curves referring to a survival probability of 50%, while all other statistical parameters have been reported in Table 2.

Table 2 evaluates the mean stress effect on the high-cycle fatigue strength of the nodular cast iron because it compares the results obtained with a nominal load ratio R = 0.05 (namely with mean stress practically equal to the stress amplitude σ_m_ = σ_a_) and the results referred to a nominal load ratio R = −1 (namely with a null mean stress σ_m_ = 0). It can be observed that the mean stress effect is strong for plain specimens, the high-cycle fatigue strength being 204 MPa for R = −1 and 124 MPa for R = 0.05, which corresponds to a reduction of 34%. On the other hand, it is significantly reduced for sharply V-notched specimens, with σ_A,50%_ being 66 and 53 MPa for R = −1 and 0.05, respectively, which translates into a reduction of 19%. The inverse slope of the fatigue curves is almost insensitive to the load ratio, being equal to 8.87 and 8.28 for plain specimens under R = −1 and 0.05, respectively, and 4.50 and 4.90 for sharply V-notched specimens under R = −1 and 0.05, respectively. Blunt notched specimens exhibited high-cycle fatigue strength and inverse slope intermediate between those of plain and sharply V-notched specimens, being σ_A,50%_ = 79 MPa and k = 5.84. In all cases, the scatter index has been rather reduced, being in the range between 1.26 (bluntly notched specimens under R = 0.05) and 1.43 (plain specimens under R = 0.05).

Figure 8 also shows that some specimens (highlighted by a red symbol ‘*’) failed prematurely compared to the average trend or to other specimens reaching run out condition even if tested at a higher load level. This behaviour can be justified by observing the fracture surfaces (Figure 7), which show that:

- The plain specimen tested at R = −1 fails from a very large sub-surface defect (see Figure 7a);
- The plain specimen tested at R = 0.05 fails from a large sub-surface defect (see Figure 7d);
- The sharply V-notched specimens tested at R = −1 and 0.05 shows multiple crack initiation locations, in addition to the notch tip, i.e., a sub-surface defect (see Figure 7f) and a graphite nodule (see Figure 7g).

On the other hand, the case of the bluntly notched specimen seems not to be justified since no defects have been observed in the fracture surface (see Figure 7i).

Finally, a master curve in terms of nominal stress amplitude versus fatigue life cannot be defined because the net-section nominal stress is not able to correlate notch geometry and mean stress effects, as the highly scattered results reported in Figure 8 demonstrate. To define a master curve, the local approach presented in the previous Section will be applied in the following Section.

### 4.3. Application of the Averaged SED Approach

**Figure 9:**
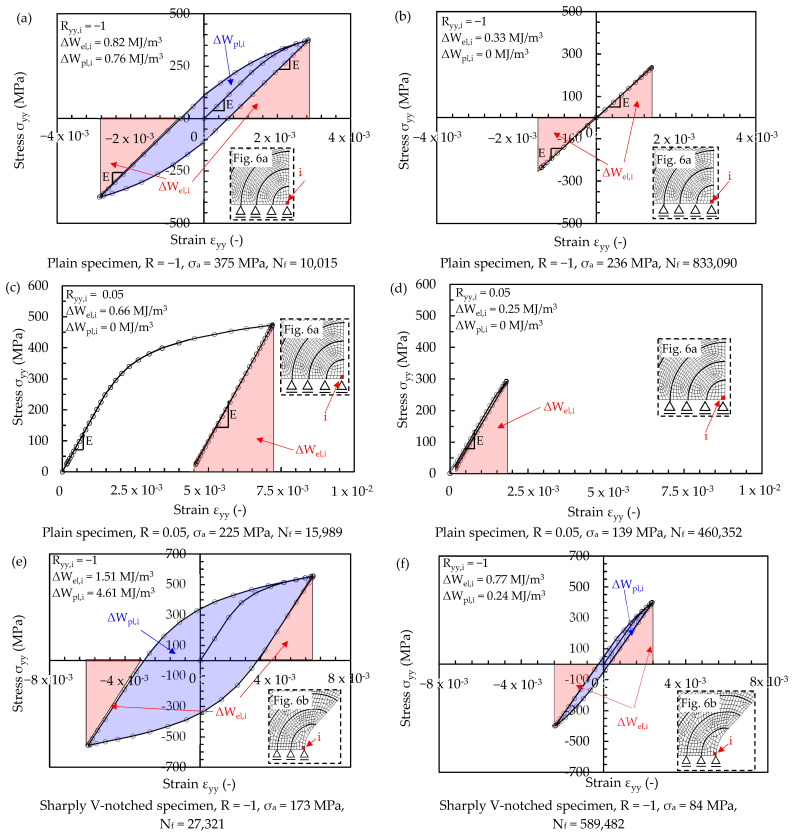

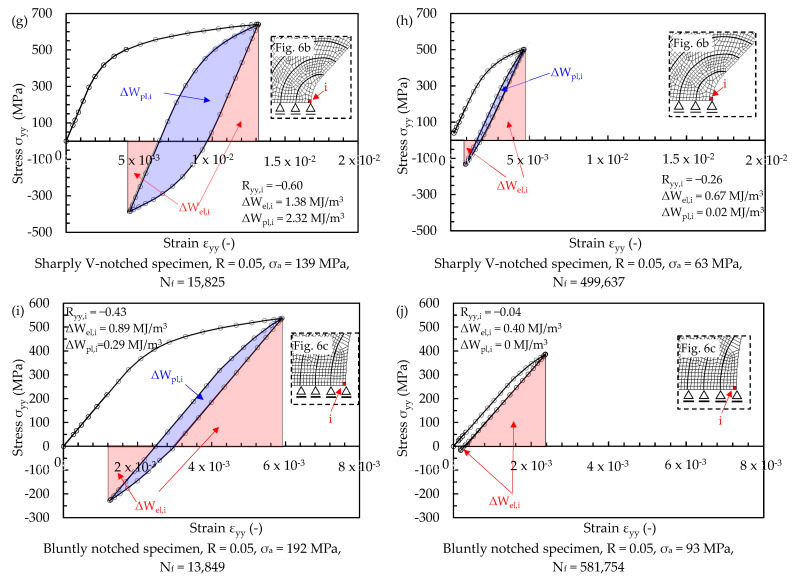
Hysteresis loops σ_yy_-ε_yy_ for plain specimens under (**a**,**b**) R = −1 and (**c**,**d**) R = 0.05; sharply V-notched specimens under (**e**,**f**) R = −1 and (**g**,**h**) R = 0.05; bluntly notched specimens under (**i**,**j**) R = 0.05. The hysteresis loops are calculated by FEM with reference to an *i*-th finite element located at the outer surface of the plain specimen and at the tip of the notched specimens (see Figure 6 and the zoom inside each figure).

After having solved the FE models shown in Figure 6, the averaged SED values have been calculated as follows:

- In the case of linear elastic material behaviour, the averaged SED has been calculated by applying Equation (4) directly in the post-processing environment of Ansys^®^, for each specimen geometry and each value of R_0_. Afterwards, the quadratic proportionality $\Delta \bar{W}\propto \Delta \sigma^{2}={\left(2\cdot \sigma_{a}\right)}^{2}$, which can be derived from Equations (1) and (4), has been exploited to calculate the averaged SED with reference to the experimental stress amplitudes. Finally, the proper coefficient c_w_ (Equation (2)), i.e., c_w_ = 0.5 and 1.1 for R = −1 and 0.05, respectively, has been applied.
- Concerning the elastic plastic material behaviour, the hysteresis loops σ_jk_-ε_jk_ have been calculated in the post-processing environment of Ansys^®^ for each specimen geometry, load case and *i*-th finite element belonging to the structural volume having radius R_0_. Then, the elastic and plastic strain energy density contributions, i.e., $\Delta W_{el,i}$ and $\Delta W_{pl,i}$, have been calculated by post-processing the hysteresis loops through a dedicated Python^®^ code. Finally, Equation (6) has been applied to compute the averaged SED. Figure 9 reports examples of the hysteresis loops σ_yy_-ε_yy_, i.e., stress and strain components acting along the loading axis (see Figure 6), for plain, sharply V-notched and bluntly notched specimens with reference to a finite element located at the outer surface of the plain specimen and at the tip of the notched specimens. Two examples of hysteresis loops have been reported for each test series, namely one referred to a medium–low number of cycles to failure, and the other to a high number of cycles to failure. The definition of elastic and plastic strain energy densities is included in Figure 9. It is worth noting that such SED contributions are the only ones present in the plain specimens, being a uniaxial loading condition, while the axial loading applied to notched cylindrical specimens generates axial and hoop stress–strain components, i.e., σ_yy_-ε_yy_ and σ_zz_-ε_zz_ (according to Figure 6). In the high-cycle cases, Figure 9 shows that the plastic contribution is null for plain and bluntly notched specimens, while it is reduced at the tip of sharply V-notched specimens. In the medium–low cycle cases, the plastic contribution is pronounced for plain specimens under R = −1, at the tip of sharply V-notched specimens under R = −1 and 0.05; then, it is reduced at the tip of bluntly notched specimens and null for plain specimens under R = 0.05. Moreover, the local stress ratio R_i,yy_ = σ_yy,min_/σ_yy,max_ is equal to the nominal one for plain specimens regardless of the load ratio (Figure 9a–d) and notched specimens under R = −1 (Figure 9e,f), while plastic deformations localised at the notch tip shift the local stress ratio R_i,yy_ to negative values when notched specimens are subjected to R = 0.05 (Figure 9g–i,j).

**Figure 10:**
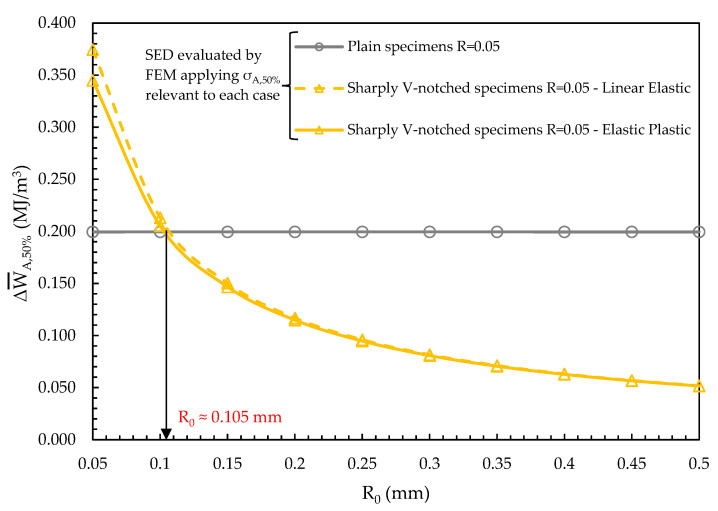
Averaged SED calculated for plain and sharply V-notched specimens with reference to the case R = 0.05, a fatigue life N_A_ = 2 × 10^6^ cycles and a survival probability 50%, equated to calibrate the structural volume size R_0_. The averaged SED of the sharply V-notched specimen configuration has been calculated by assuming both a linear elastic and an elastic plastic material behaviour.

Afterwards, the calibration of the structural volume size, R_0_, has been performed by equating the averaged SED calculated for plain and sharply V-notched specimens tested at R = 0.05 with reference to a fatigue life N_A_ = 2 × 10^6^ cycles and a survival probability 50%. The averaged SED of the plain specimen has been calculated by assuming a linear elastic material behaviour, the plastic contribution being null as demonstrated in Figure 9c,d. On the other hand, the averaged SED of sharply V-notched specimen has been calculated by assuming both a linear elastic and an elastic plastic material behaviour. The results in terms of $\Delta {\bar{W}}_{A,50\%}$ versus R_0_ are reported in Figure 10, which shows that $\Delta {\bar{W}}_{A,50\%}$ assumes the same value for both specimen configurations and material behaviours if the structural volume size is R_0_ = 0.105 mm. Figure 10 shows also that the averaged SED of the sharply V-notched specimen under R = 0.05 slightly differs when calculated under linear elastic or elastic plastic material behaviour only for reduced values of R_0_, i.e., close to the notch tip where plastic deformations are localised. This was expected also on the basis of Figure 9h, which shows that the plastic SED contribution is reduced even for a fatigue life of N_f_ = 0.5 × 10^6^ cycles.

**Figure 11:**
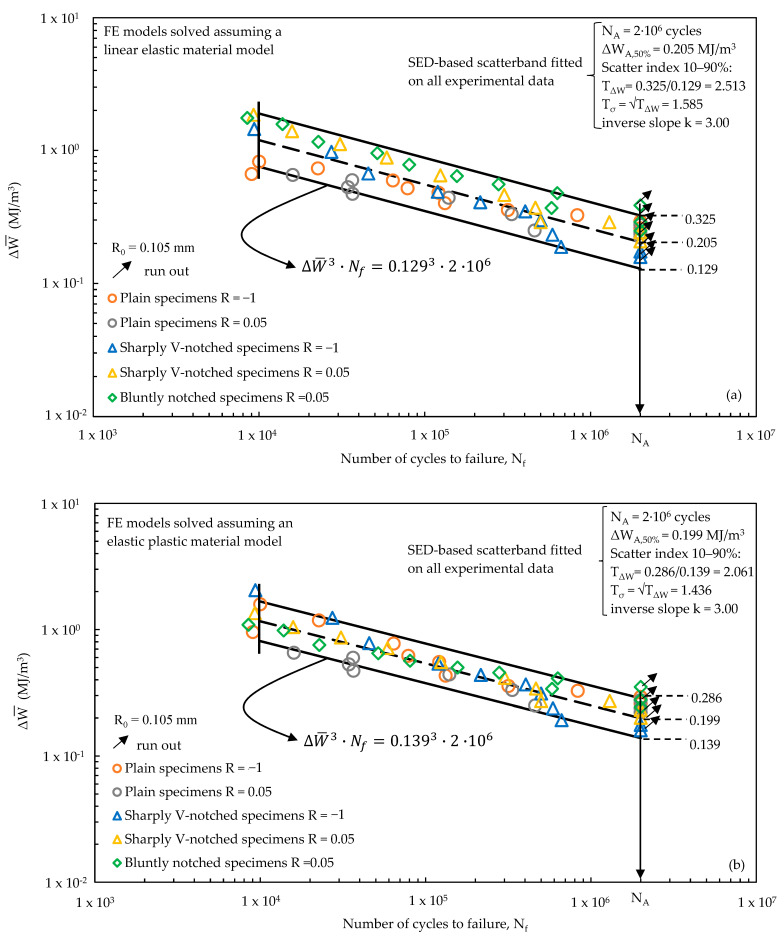
Averaged SED-based scatter-bands fitted on the experimental results reported in terms of number of cycles to failure as a function of the SED averaged over a structural volume having size R_0_ = 0.105 mm. The averaged SED was computed by assuming (**a**) a linear elastic and (**b**) an elastic plastic material behaviour. The figures include also the expression of the design curves referred to a probability of survival of 90%, which allow to derive the fatigue life once the strain energy density has been calculated.

Afterwards, the experimental fatigue results reported in Figure 8 and expressed in terms of nominal stress amplitude σ_a_ (Equation (7)) have been converted to the SED $\Delta \bar{W}$ averaged over a structural volume having size R_0_ = 0.105 mm. Figure 11 plots $\Delta \bar{W}$, calculated by assuming a linear elastic (Figure 11a) or an elastic plastic (Figure 11b) material behaviour in the FE analyses, against the experimental number of cycles to failure N_f_.

A statistical analysis has been performed to derive the average SED-based scatter bands in the range of number of cycles between 10^4^ and 2∙10^6^. Figure 11 shows that in both cases the inverse slope k results equal to 3; however, the comparison with Figure 11a,b clearly highlights that $\Delta \bar{W}$, calculated assuming an elastic plastic material behaviour, is capable of better synthesis of the experimental data. In fact, Figure 11a shows that the experimental data of each test series are well separated and distinguishable, even if summarised in a single quite-narrow scatter band. On the other hand, Figure 11b shows that the experimental results are mixed as belonging to a single test series. This is also confirmed by the equivalent stress-based scatter index T_σ_ = √T_W_, which is equal to 1.585 and 1.436 for the linear elastic and elastic plastic SED-based synthesis, respectively.

**Figure 12:**
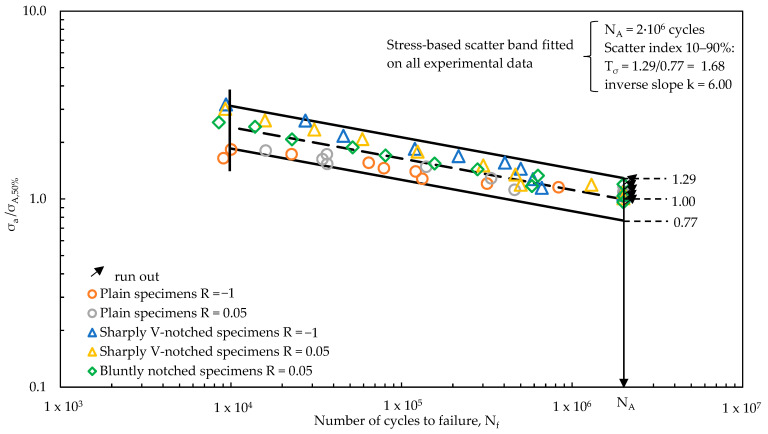
Calibration of the intrinsic scatter-band on the experimental fatigue data of Figure 8 normalised by means of the relevant σ_A,50%_, as reported in Table 2.

The effectiveness of SED-based synthesis has been furtherly evaluated by analysing the intrinsic scatter of the experimental results. To do this, the experimental results have been statistically re-analysed by excluding the different notch and mean stress effects; therefore, a normalisation of the data of each test series has been performed by means of the relevant high-cycle fatigue strength σ_A,50%_, as reported in Table 2. Figure 12 reports the normalised scatter-band, having an intrinsic scatter, referred to 10–90% probability of survival, equal to T_σ_ = 1.68.

Finally, Figure 11b suggests that the elastic plastic formulation of the averaged SED approach, providing a stress-based scatter index equal to 1.436 and lower than the intrinsic scatter of 1.68, effectively summarises the various notch effects and mean stress effects on the fatigue strength of EN-GJS-500-7 cast iron. Therefore, the master curve defined in Figure 11b may be used for the fatigue design of structural components, for example, off-highway axles, provided that they are made of EN-GJS-500-7 nodular cast iron having the microstructure shown in Figure 4. The master curve is applicable regardless of the geometry and the loading condition since both notch geometry and mean stress effects are accounted for in the averaged SED fatigue damage parameter.

For the sake of completeness, it is worth noting that the following assumptions have been made in the averaged SED approach applied in the present investigation:

- *Material in FE analyses*: a homogeneous, multi-linear kinematic hardening material model was assumed. Alternatively, a Chaboche non-linear isotropic kinematic hardening model [58] can be employed to capture the material behaviour evolution during cyclic loading; however, calibrating such a model is rather complex and typically requires material strain-based fatigue data using different strain amplitudes and rates.
- *Material microstructure*: the SED-based master curve reported in Figure 11b is valid for a nodular cast iron EN-GJS-500-7 having the microstructure reported in Figure 4.
- *Crack initiation location*: the SED has been averaged over a structural volume lying on the outer surface of a plain specimen and embracing the notch tip of sharply V-notched and bluntly notched specimens (see Figure 6); therefore, it has been assumed that the structural volume having size R_0_ accounts for the material microstructure, including discontinuities such defects and graphite nodules. This assumption is supported by Figure 8, which shows a reduced scatter index of each test series even if the crack initiation occurred either at the specimen surface or from sub-surface defects for plain specimens, either from the notch tip or from defects or graphite nodules for notched specimens.

## 5. Conclusions

In this study, the effects of notches and mean stresses on the fatigue behaviour of EN-GJS-500-7 nodular cast iron, adopted in a real off-highway axle, have been examined. To this aim, laboratory specimens, having plain, sharply V-notched and bluntly notched cylindrical geometries, have been extracted from an off-highway axle to take into account the manufacturing process effects. Load-controlled axial fatigue tests were conducted by using two nominal load ratios, namely R = −1 and R = 0.05. The following conclusions can be drawn:

- The fracture surfaces analyses performed by SEM microscope showed that in plain specimens, the fatigue crack initiated either from the surface or sub-surface defects. On the other hand, in sharply V-notched and bluntly notched specimens, fatigue crack initiation mainly occurred from the notch tip, with additional crack initiations having been observed from sub-surface defects and graphite nodules.
- The experimental fatigue results of each test series were presented in terms of the number of cycles to failure N_f_ as a function of the nominal net-section stress amplitude σ_a_. Plain specimens showed a strong mean stress effect, with the high-cycle fatigue strength σ_A,50%_ being 204 MPa (k = 8.87) for R = −1 and 124 MPa (k = 8.28) for R = 0.05, i.e., 34% lower for higher mean stress (R = 0.05). On the other hand, sharply V-notched specimens exhibited a reduced mean stress effect, with σ_A,50%_ being 66 MPa (k = 4.50) and 53 MPa (k = 4.90) for R = −1 and 0.05, respectively, i.e., 19% lower for higher mean stress (R = 0.05). Bluntly notched specimens have been tested only under R = 0.05 and exhibited σ_A,50%_ = 79 MPa (k = 5.84). In all cases the 10–90% scatter index T_σ_ was rather reduced, being in the range between 1.26 and 1.43.
- Finally, the fatigue approach based on the strain energy density (SED) averaged over a properly defined structural volume has been applied. The structural volume size R_0_ has been calibrated and is equal to 0.105 mm. FE analyses have been performed by assuming either a linear elastic or an elastic plastic material behaviour to convert the experimental fatigue results from the nominal net-section stress amplitude to the averaged SED value. The elastic plastic formulation of the averaged SED approach has shown to be able to well correlate the different notch effects and mean stress effects on the fatigue strength of EN-GJS-500-7 cast iron. Therefore, the relevant master curve can be useful for the fatigue design of off-highway axles made of EN-GJS-500-7 cast iron.

## Figures and Tables

**Table 1:** Static tensile and strain-controlled fatigue properties of EN-GJS-500-7 as derived in [51].

	Static Tensile Curve							Stabilised Cyclic Curve	
Material	E	σ_p0.2_	σ_R_	A	Z	K	n	K′	n′
	[MPa]	[MPa]	[MPa]	[%]	[%]	[MPa]	[/]	[MPa]	[/]
EN-GJS-500-7	170,400	360	608	9.25	8.75	902	0.157	927.6	0.123

**Table 2:** Summary of load-controlled axial fatigue test results.

Material	Specimen Geometry *	d_gross_ (mm)	d_net_ (mm)	ρ (mm)	2α (°)	R	N° Data	N° Runout	σ_A,50%_ (MPa)	k	T_σ_
EN-GJS-500-7	Plain	10	5.5	-	- -	−1 0.05	11 10	2 3	204 124	8.87 8.28	1.35 1.43
	Sharply V-notched	10	5.5	0.1	90	−1 0.05	11 11	2 2	66 53	4.50 4.90	1.41 1.33
	Bluntly notched	10	5.5	0.8	90	0.05	12	3	79	5.84	1.26

*—plain specimen (Figure 5a), sharply V-notched specimen (Figure 5b) and bluntly notched specimen (Figure 5c).

## Data Availability

All data are contained within the present article.
